# Effective Reduced Diffusion-Models: A Data Driven Approach to the Analysis of Neuronal Dynamics

**DOI:** 10.1371/journal.pcbi.1000587

**Published:** 2009-12-04

**Authors:** Gustavo Deco, Daniel Martí, Anders Ledberg, Ramon Reig, Maria V. Sanchez Vives

**Affiliations:** 1 Institució Catalana de Recerca i Estudis Avançats (ICREA), Barcelona, Spain; 2 Computational Neuroscience Group, DTIC, Universitat Pompeu Fabra, Barcelona, Spain; 3 Institut d'Investigacions Biomèdiques August Pi i Sunyer (IDIBAPS), Barcelona, Spain; University College London, United Kingdom

## Abstract

We introduce in this paper a new method for reducing neurodynamical data to an effective diffusion equation, either experimentally or using simulations of biophysically detailed models. The dimensionality of the data is first reduced to the first principal component, and then fitted by the stationary solution of a mean-field-like one-dimensional Langevin equation, which describes the motion of a Brownian particle in a potential. The advantage of such description is that the stationary probability density of the dynamical variable can be easily derived. We applied this method to the analysis of cortical network dynamics during up and down states in an anesthetized animal. During deep anesthesia, intracellularly recorded up and down states transitions occurred with high regularity and could not be adequately described by a one-dimensional diffusion equation. Under lighter anesthesia, however, the distributions of the times spent in the up and down states were better fitted by such a model, suggesting a role for noise in determining the time spent in a particular state.

## Author Summary

We introduce a novel methodology that allows for an effective description of a neurodynamical system in a data-driven fashion. In particular, no knowledge of the dynamics operating at the neuronal or synaptic level is required. The idea is to fit the underlying dynamics of the data using a stochastic differential equation. We use a Langevin equation that describes the stochastic dynamics of the system with the assumption that there exists an underlying potential, or energy function. The advantage of this description is the fact that, for one-dimensional systems, the stationary distribution of the variable can be straightforwardly related to the underlying energy function. In cases where the dataset is high-dimensional we reduce the dimensionality with techniques like principal curves or principal components analysis. The methodology we propose is particularly relevant for cases where an *ab initio* approach cannot be applied like, for example, when an explicit description of the dynamics at the neuronal and synaptic levels is not available.

## Introduction

Deciphering the fundamental mechanisms that underlie brain function requires an explicit description of the dynamics of the neuronal and synaptic substrate. Explicit neurodynamical models can describe the complex dynamics arising from the involved neuronal networks [1],[2]. Traditionally, theoretical neuroscience follows an *ab initio* approach consisting of two main steps: 1) construction and simulation of models based on detailed descriptions of the neuronal and synaptic operations with a large number of neurons in a specified (hypothesized) network architecture, and 2) reduction of the hypothesized models such that an in-depth analytical study is feasible, and a systematic relation between structure (parameters), dynamics, and functional behavior can be solidly established. Models of neurons such as integrate-and-fire [3] are frequently used. The advantage of this type of models is that the simulation of biologically realistic networks allows the study of the neural correlates of brain function, for comparison with experimental data. On the other hand, the model is simple enough so that it is possible to obtain a reduced description based on *mean-field* techniques [4],[5]. The mean-field reduction simplifies the analysis of networks of spiking neurons, by partitioning the network into populations of neurons that share the same statistical properties. Using some plausible approximations, the stationary firing rate of each population can be expressed as a function of the firing rates of all the populations in the network. The set of stationary, self-reproducing rates for the different populations in the network can then be found solving a set of coupled self-consistency equations (see e.g. [4]). The method allows to characterize the activity of the network as a function of the neuronal and synaptic parameters.

For this *ab initio* approach to be applicable, however, one needs an explicit representation of the dynamics at the microscopic level. Even when such representation is actually available, it may not be possible or easy to come up with a low-dimensional description of the original system. Here we introduce an alternative methodology that allows for an effective reduction of dimensionality. The method is data-driven, in the sense that it does not require any knowledge of the dynamics at the microscopic level. The basic idea is is to fit the underlying dynamics of the data using a stochastic, nonlinear differential equation. In general, fitting a model to data from a nonlinear stochastic system is a difficult problem because of the high dimensionality of the space of available models. Without some prior knowledge to guide model selection, the likelihood of picking the “correct” model for some data set is slim. Here we describe a method that can be applied to data from systems that are (a) stationary, (b) driven by additive white noise, and (c) whose deterministic motion is governed by an effective one-dimensional energy function. In such type of systems, the stationary distribution of the variable can be straightforwardly related with the underlying energy function. When neurodynamical data is high-dimensional, the dimensionality of the system can be first reduced using principal curves or principal components analysis.

To model data from such systems we proceed in two steps. We estimate first the energy function and then the intensity of the noise. The energy function is uniquely determined by the stationary distribution, so to accomplish the first step we estimate this distribution from data. In particular we assume that we have access to samples from this distribution and that the underlying potential can be fit by a piecewise quadratic polynomial. To fit the intensity of the noise we need a measure that is dependent on this parameter in a known way. In this work we use the mean first-passage time through a particular boundary, for which there are closed-form expressions. That is, given samples of the first-passage times of the system under study, we find the noise intensity that yields the same mean first-passage time in the system described by the fitted energy function.

We first apply the method to simulated data from a one-dimensional rate equation. In this case, the assumptions of the method are fulfilled and we can recover the original model with high accuracy if the number of data points is sufficiently high. Next we show that the method is applicable also to data from high-dimensional neuronal models. In particular, we use the method to obtain the effective dynamics of a network of spiking neurons operating near a bifurcation. Finally, we apply the method to real data from intracellular recordings from cortical neurons *in vivo*.

## Results

We next show the effectiveness of the method by applying it to three different systems of different complexity: (1) a one-dimensional stochastic rate model; (2) a network of spiking neurons showing bistability; and (3) experimental data from slow oscillatory activity in the cerebral cortex *in vivo*.

### One-dimensional rate model

Here we show how the method works for a one-dimensional rate model described by a Langevin equation,
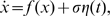
(1)where 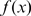 is a nonlinear function and 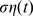 is Gaussian white noise with standard deviation 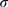. The method described in this article provides an effective description of the system of exactly the same type as Equation (1), given a sample of states 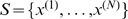. Since the system is one-dimensional, an energy function 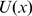 satisfying 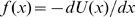 can be trivially defined without resorting to any approximation method. Thus, we do not gain much insight in applying this method to such a simple system. Our aim in this section is rather to check that the piecewise approximation of the probability density described in Methods recovers correctly the energy of the system, which is well-defined in this particular example. We also study the sensitivity of the estimation to the number of subintervals used in the piecewise quadratic approximation.

#### Fixed points and stability of the noiseless system

For the sake of concreteness, we choose 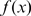 to be of the form:
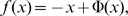
(2)where 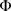 is a sigmoidal activation function
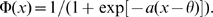
(3)The parameter 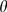 sets the location of the sigmoid's soft threshold, and 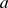 is a scale parameter. This specific choice of 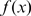 is motivated by the form of the rate models used to describe the firing activity of neuronal assemblies [6]–[8], although any other system satisfying the condition 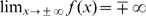, which ensures the existence of at least one stable fixed point, would be equally valid (see Methods).

**Figure 1:**
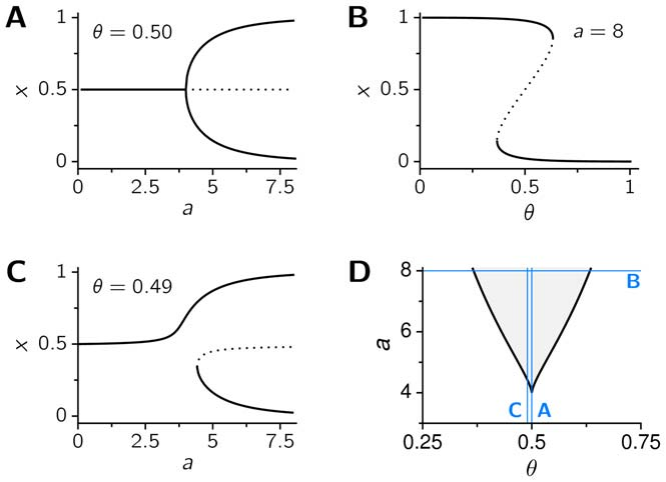
Bistability in a one-dimensional rate model. The rate model described by Equations (1)–(3) has two stable fixed points for some values of the parameters 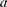 and 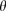. A, B, and C: Location of the fixed points as a function of 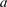, for 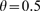 (A) and 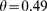 (C), and as a function of 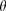, with 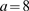 (B). Solid curves correspond to stable fixed points, and the dotted curves to unstable fixed points. D: Regions of monostability (white) and bistability (gray) in the parameter space 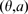. Blue lines show the sections of the parameter space represented in the bifurcation diagrams A–C.

We first consider the deterministic system that results from switching noise off by setting 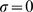 in Equation (1). The fixed points of the noiseless system satisfy the condition 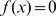 or, equivalently, 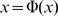. Depending on the values of the parameters 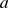 and 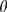, there may be either one or three solutions to this equation. In the former case, the only intersection point will always correspond to a stable fixed point by construction, and the system will be monostable. When instead three solutions coexist, the system is bistable, with two of the solutions corresponding to stable fixed points and the remaining one corresponding to an unstable fixed point. We illustrate in Figures 1A–C the presence and number of fixed points as a function of the parameters. Note that bistability is only possible for high enough values of 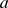, which dictates the degree of nonlinearity in the system. Also, in order for the system to be bistable, the value of 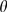 should lie in some interval centered at 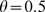. The endpoints of the interval can be actually derived from the fixed point condition, 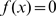, and the condition 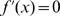 that determines where pairs of fixed points appear or disappear (see Text S1). The region of bistability shown in Figure 1D.

It is straightforward to compute the energy function of the system:
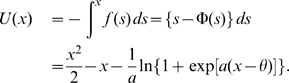
(4)By construction, the local minima of the energy function 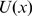 are at the stable fixed points of the system (1), while the local maxima are at the unstable fixed points.

#### Stochastic system: extraction of the effective parameters

A bistable deterministic system like the one described in the previous section will always decay to either one of the two available stable states depending on the initial conditions. Once reached equilibrium, the system will remain there indefinitely. The picture changes dramatically when noise is added to the system: the variable 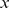 spends most of the time wandering around either one of the two fixed points until some rare and large fluctuation drives it to the neighborhood of the other fixed point, where the process starts over. Therefore, rather than a pair of stable states, we have two *metastable* states that are long-lived in terms of the characteristic time scales of the system, but that are not truly stable at much longer time scales. Each of these two metastable *states* are better regarded as a unimodal *distribution* centered at one of the stable fixed points of the noiseless system. We loosely refer to each of these distributions as an *attractor*. Since the system spends most of the time in either one of the attractors, and there are quick, random, and rare switches between the two, the stationary distribution of 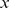 is bimodal.

**Figure 2:**
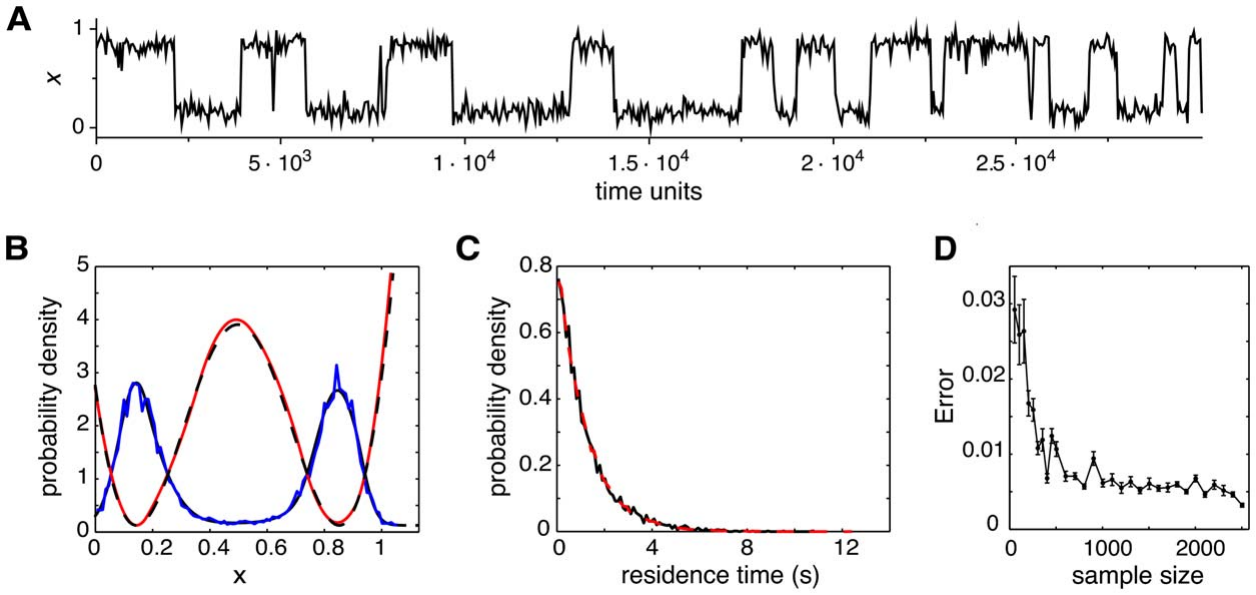
Statistical properties of the one-dimensional, bistable rate model in the presence of noise. A: Stationary distribution of the rate variable 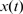 of the system (1)–(3) with parameter values 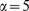, 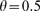, 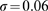. Blue: normalized histogram of the simulated data. Black: maximum likelihood fit of the stationary distribution, using a piecewise quadratic approximation. Red: estimated energy function. Black dashed: original energy function. B: Distribution of the residence times in each of the attractors. Due to the symmetry of the system when 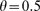, the two attractor states share the same statistical properties; here we show only the distribution of residence times in the *left* attractor, 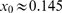. Red and black curves are, respectively, the distributions estimated from the original stochastic Equation (1) and from the reconstructed system. C: Error in the estimation of the true energy function, as a function of the number of data points. Error bars are mean squared errors.

As an example, we choose the parameter values 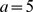 and 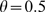, which lie within region of bistability depicted in Figure 1D. The analysis of stability of the deterministic limit of Equations (1)–(3) shows that for 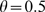 and 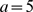 the stable fixed points are located at 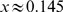 and 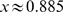. By adding a moderate amount of noise, the two stable fixed points become metastable and alternate with each other (Figure 2A). The stationary distribution of the state variable 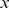, when the stochastic system described by Equations (1)–(3) is simulated long enough, is shown in Figure 2B. Note that we are implicitly assuming that the sampling of the system over a long enough time can be identified with the sampling of independent realizations of the same process —i.e., we are assuming ergodicity. Figure 2B also shows the maximum likelihood estimate of the stationary distribution 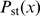 using a piecewise quadratic approximation, as well as the associated energy function. The two peaks of the distribution are centered at the stable fixed points of the noiseless system. With the estimated probability density we can easily extract the underlying energy, 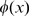, following the procedure described in Methods. For a comparison with the estimated energy function, we also include in Figure 2B the true energy function of the original system (Equation (4)). Note the good agreement between the two.

The noise intensity was estimated from the mean escape time from a metastable state, Equation (13). In our example, we estimated the average time needed for the system initialized at the fixed point at 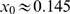 (*left* attractor) to cross a boundary at some 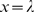. The location 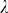 of the boundary was chosen somewhere between the separatrix at 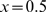 to the fixed point at 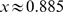 (*right* attractor), to make sure that the mean first-passage time corresponded to a real escape from one attractor to the other. The analytical form for the mean escape time from an interval 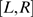, when the system is initialized at 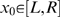, is given by Equation (13) in Methods. The expression (13) can be further simplified using the property that 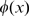 goes to infinity in the limit 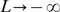, which ensures the system can escape only through the right endpoint of the interval. Thus identifying 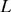 with 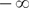 and 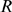 with 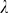, the mean escape time through 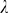 reads
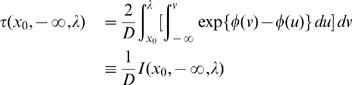
(5)The noise intensity can then be estimated as 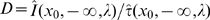, where 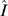 is the numerical evaluation of 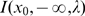 using the piecewise-quadratic approximation of the potential, and 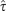 is the sample mean of first-passage times. We obtained an estimated value for the noise intensity of 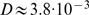, which is close to the value 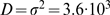, used in the simulations. The value of the error in estimating the true energy function, as a function of the number of data points, is shown in Figure 2D. Good fits are already obtained with 300 data points.

### Network of spiking neurons

**Figure 3:**
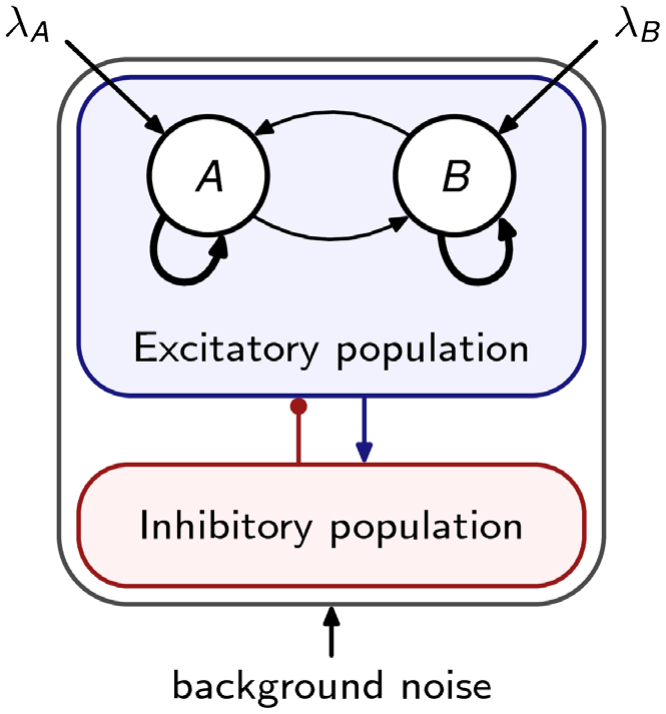
Architecture of the winner-take-all spiking network. The network is fully connected and structured in different subpopulations of cells sharing the same connectivity and input statistics. All neurons receive background noise input modeled as independent Poisson trains. Cells in neural populations 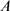 and 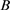 receive in addition a Poisson train of rate 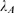 and 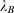, respectively, to account for selective input. These two so-called selective populations are composed of excitatory cells strongly interconnected.

As a second example of our reduction method, we consider a large-scale network of spiking neurons exhibiting bistability. The network we use is the binary decision network introduced by Wang [9], with identical architecture and parameters (see Figure 3 and Text S1 for the details).

In short, the model consists of a fully connected network of integrate-and-fire neurons with synaptic dynamics mediated by excitatory AMPA and NMDA receptors, and by inhibitory GABA receptors [4]. Excitatory neurons are structured into two subpopulations. Due to the strong recurrent connections between cells within each population and to the shared inhibitory feedback, the two subpopulations compete with each other for higher activity. This competition eventually culminates with the network settling into an attractor where the activation of one population suppresses the activity of the other. There are two such attractors, called asymmetric attractors, associated with the two possible outcomes of the competition. Apart from recurrent currents, all cells receive AMPA-mediated synaptic currents from external neurons that emit spikes following Poisson statistics.

**Figure 4:**
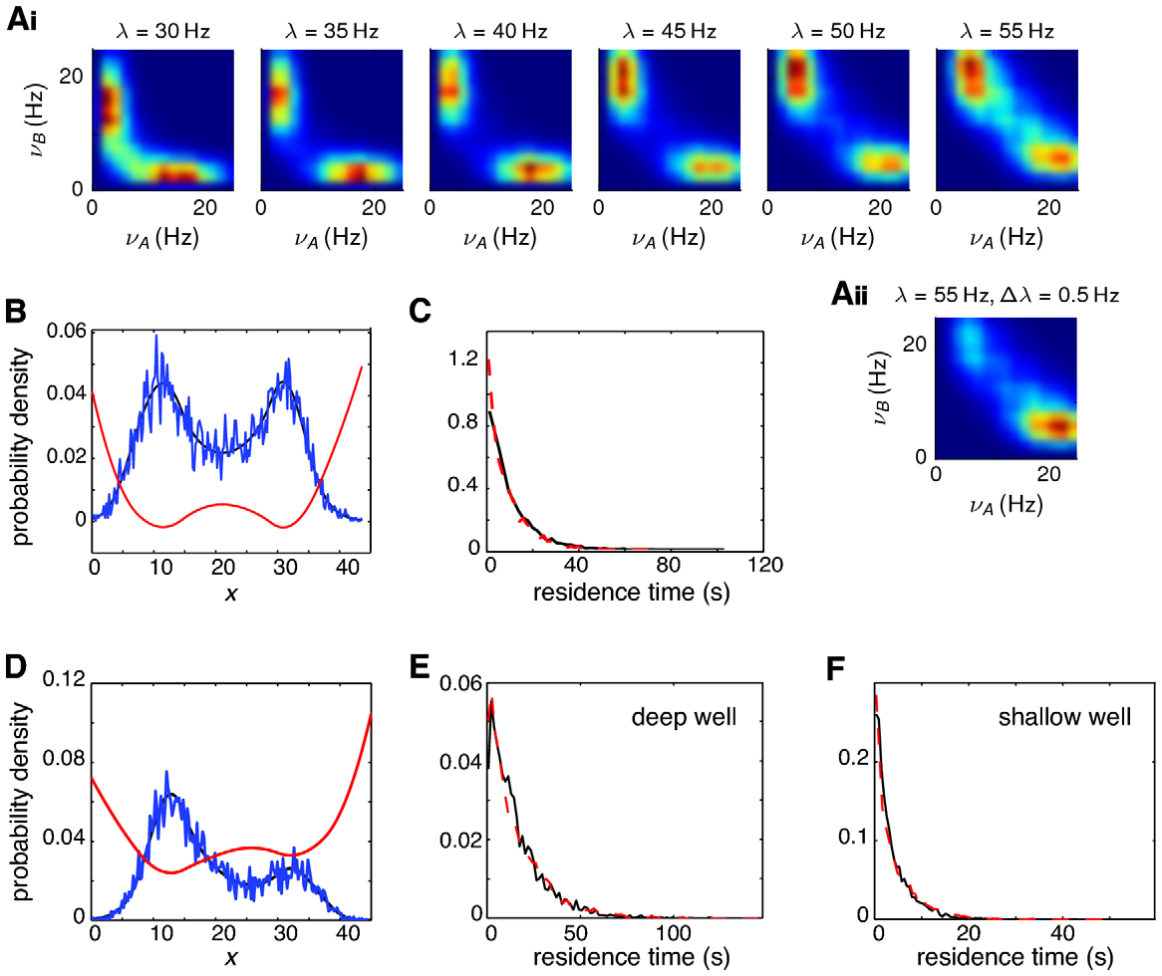
Extraction of the effective parameters from data generated by a winner-take-all network of spiking neurons. A: Estimated probability density functions of the population rates 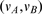. (Ai): symmetric network with balanced external inputs (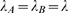) for different values of input intensity 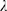, indicated at the top of each plot. Aii: unbalanced inputs, 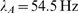, 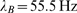. Probability densities are shown as 2-dimensional histograms of 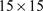 bins and Gaussian interpolation. B, and D: Blue: stationary distribution of the projection on the principal component 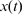 of the firing rate 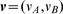 of a network with symmetric (B) and asymmetric (D) inputs. Black: maximum likelihood fit using a piecewise quadratic approximation. Red: energy function. C, E, F: Distribution of the residence times in the attractor states, for the symmetric (C) and asymmetric cases (E,F). For the asymmetric case, the *deep* attractor corresponds to the network state where the active population firing at highest rate is the population receiving strongest inputs. Conversely, in the *shallow* attractor the active population is that receiving weakest inputs. The dashed red curves are the distributions estimated directly from the data, while the solid black curves are the distributions derived from the effective one-dimensional Langevin system.

For a wide range of external inputs and connection weights, the network operates as a winner-take-all, and is therefore able to sustain either one of the two asymmetric stable states. As in the rate model analyzed in the previous section, noise induces transitions between states that are simultaneously stable, giving rise to a bimodal distribution in the rate variables when the system is observed long enough. This bimodality can be seen in Figure 4A, which shows the two-dimensional histograms of the population-averaged activities of both populations, for different levels of external input.

Note that the strength of the method is not in reducing the dimensionality of the system, but in extracting effectively the underlying stochastic dynamics in the form of a diffusion equation. Thus a prerequisite for applying the method is to select a range of parameters where the dynamics of the system can be reduced to one-dimensional dynamics. In this type of system, this is the case in the neighborhood of a bifurcation (see, e.g., [10]).

Given the reduced first principal component 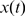 of the original data, we apply the procedure detailed in Methods to extract the effective energy function associated with a one-dimensional Langevin equation (see Equations (6) and (9)). We show in Figures 4B and 4D the effective energy function for the symmetric and asymmetric case, respectively. The figures also show the stationary distribution of the reduced variable 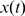 capturing the essential part of the dynamics, as well as the maximum likelihood fit of 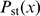, Equation (10). By using Equation (10) we can easily extract the effective energy function 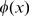.

We then estimated the noise intensity along the same lines of the previous section. In brief, we generated a large set of sample paths, starting out at one the attractors, and computed the first-passage time through some prescribed boundary. In our case, the boundary was halfway to the main barrier separating the two attractors. Choosing the boundary this way, the first-passage times were considerably shorter than the transition times between attractors, allowing for larger samples and thus better numerical estimation. We could then estimate from Eq.(13) the noise intensity, using the sample mean of the first-passage times and the effective potential. For the symmetric case (Figures 4B,C), we initialized the system at 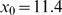 and set the boundary at 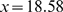. For the asymmetric case (Figure 4D–F), the system was initialized at 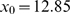 and the barrier was at 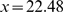.

Using the estimates of the noise intensity 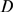 and the effective energy function 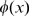, we checked the approximation by comparing the residence times in each of the attractors (Figures 4C and 4E,F for the symmetric and asymmetric case, respectively). The agreement between the distribution of residence times for the one-dimensional Langevin and for the original data is remarkable.

Note that with this method we can easily estimate the transition times between attractors, using just the one-dimensional reduced system. This is particularly useful when the transition times are long, of the order of seconds, for which a reliable estimation requires simulations of the high-dimensional system, defined in our case by a system of several thousands of nonlinear differential equations. The reduction can be done without such computational effort, since it requires only a good estimate of the stationary distribution, to extract the underlying energy, as well as an estimate of the escape times, in order to get the estimate of the noise intensity. The effective data-driven reduction allows us to extract explicitly the underlying form of the energy function associated with the bistable behavior, the level of fluctuations, and consequently allow us to calculate the characteristic escape times in a much more efficient way, due to the fact that with the reduced system they can be calculated semi-analytically.

### Up and Down State Dynamics in the Cerebral Cortex *in vivo*

**Figure 5:**
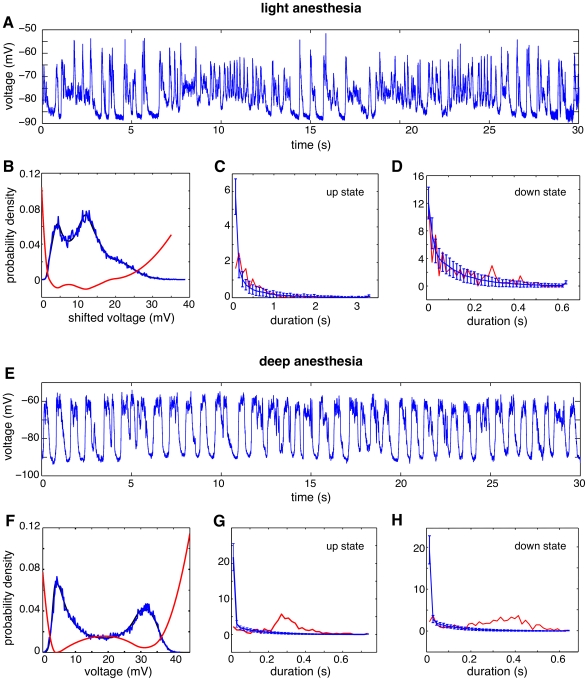
Up and down alternations recorded in vivo in the auditory cortex of a rat under light and deep anesthesia. A, and E: Trace of the subthreshold membrane potential, measured intracellularly for light (A) and deep (E) anesthesia. B, F: Stationary distribution of the membrane potential 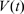 for light and deep anesthesia. Red: energy function derived from the distribution. Black: maximum likelihood estimate. C, D, G, H: Distribution of up-state and down-state durations, for light (C,D) and deep (G,H) anesthesia. Dashed red: experimental data. Solid black: data from simulations.

We next analyzed experimental data from intracellular recordings in the auditory cortex of anesthetized rats. During anesthesia with ketamine-xylazine, the cerebral cortex exhibits robust slow oscillatory activity, as it has been previously described in the cat [11],[12], ferret [13], and rat [14],[15]. Up and down states were recorded both by means of local field potential (not shown) and intracellularly (Figure 5) during periods of lighter and deeper anesthesia. Anesthesia levels were deeper after the injection of supplemental doses (see Methods).

During periods of light anesthesia, without reaching the transition to the awake state (for a complete transition from sleep to awake, see [16]), cortical activity still shows up and down states, but their distribution appears to be more random (Figure 5A). Given the normalized and centered membrane potential 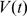 of the recorded data, we applied the procedure detailed in Methods to extract the effective energy function associated with a reduced Langevin equation, (Figure 5B). Using Equation (10) we can easily extract the underlying energy or potential function 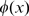. In both cases we shifted the variable 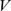 to be in the positive range, and scaled the energy function by a factor 1/100 to facilitate its visualization. We then estimated the underlying noise by using Equation (13) and the estimate of the escape time from a meta-stable state. In our case, we took the escape time that the system initialized in the down state (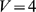) need to cross a barrier at 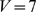. We found that the time spent in the down-state could be well fitted with our model, whereas the time spent in the up-state was less well described (Figure 5C and D).

As a quantitative measure of how well the reduced model can describe the distribution of transition times we used the Kolmogorov-Smirnov test. This is a non-parametric test of the hypothesis that two sets of observations are sampled from the same probability distribution. We applied this test to the dwell times in the down and up-states respectively (e.g. the data shown in Figure 5C and D). We can not reject the hypothesis that the dwell times in the data have the same distribution as those in the reduced model (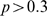). However, the distributions of the dwell-times in the upstate are significantly different (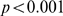). The Kolmogorov-Smirnov test hence reinforce the conclusions drawn by looking at Figure 5C and D.

During periods of deep anesthesia, up and down states generated in the cortex were quite regular, both in their amplitude and time intervals between up states (Figure 5E). Next, we will see how well this data can be described by our reduction. In this case, to estimate the noise intensity we considered the mean escape time needed for the system initialized in the down state (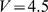) to cross a barrier at 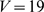. In this case, the stationary distribution can be of course fitted, but the distributions of the residence time in the down and up states cannot be captured by our model (Figures 5G and H). An application of the Kolmogorov-Smirnov test in this case confirms that dwell-times in both the up- and down-state were significantly different between the data and the reduced model (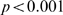 for both). In summary, when the same procedure is carried out, we no longer get a good fit of the distribution of dwell times. Note the strong regularity in the data as evidenced by the peak in the probability distribution of the life time of the experimental data. This result is nevertheless relevant because tells us that the data is not purely noise driven. In fact, previous studies have shown that these data evidenced a strong adaptation effect [17]–[20], which plays a crucial role in the transitions. The method is therefore also useful to reject the hypothesis of a pure noise driven transition.

## Discussion

We have introduced a novel methodology for extracting, in a data-driven fashion, the stochastic dynamics underlying experimental or simulated neuronal data. The main idea of the method is to test the hypothesis that the underlying dynamics is consistent with a Langevin equation, which describes the motion of a Brownian particle in a potential. This is done by extracting an effective potential consistent with the asymptotic stationary distribution of the data and subsequently estimating the intensity of the underlying fluctuations from the average escape time from a specific region. The initial hypothesis can then be tested by checking how well the escape-time distribution of the data can be fitted by the reduced model. If this fit is reasonably good then we can affirm that the observed dynamics are consistent with an underlying stochastic process of the Langevin type. Note that the test could be extended to all possible escape times, i.e., considering different escape boundaries, allowing for a sharper test of the original hypothesis. If the distribution of escape times is not well fitted by the reduced model, we can reject the hypothesis that the system is described by a one-dimensional Langevin equation.

We applied the method to data from models of simulated neuronal activity as well as recordings from real cortical neurons. The method is however applicable to data from any system that obeys the assumptions of the dynamics and the noise. Indeed, our method generalizes a similar approach suggested in the context of laser dynamics [21]. In this work we propose an efficient and semi-analytical way of estimating the noise through the estimation of Â transition times. After extracting a parametric form of the underlying energy function, we can express the transition times by a closed form expression (Equation (13)), which can be used to estimate the noise intensity. Furthermore, we use a more robust maximum-likelihood-based method for the estimation of the underlying effective energy by decomposing the stationary distribution of the main variable as a mixture of Gaussians. The method is directly applicable to data from one-dimensional systems but we also demonstrated how it could be applied to data originating from a higher dimensional system. This implied first reducing the dimensionality by projecting the data onto the first principal component, and then provide an effective model for the reduced one-dimensional data. This approach will work whenever the dynamics of the original system is confined to a one-dimensional manifold which is approximately the case for the spiking network that we studied [10],[22]. We have applied the method to data from bistable systems but it is equally straightforward to apply the method to multistable systems. As long as the dynamics can be described approximately as a diffusion in an energy landscape our method is applicable.

The fact of transitions between up and down states in the cerebral cortex is a neural network phenomenon that has aroused great interest, since the mechanisms involved may be critical for persistent activity, memory or attention. However, the cellular and network mechanisms involved in the initiation, maintenance and termination of up states are still a matter of debate. Different mechanisms of initiation of up states have been proposed, either appealing to stochastic or alternatively deterministic processes. The cortical network *in vivo* generates slow rhythmic activity in complex interaction with other rhythms in the thalamocortical network rhythmic activity [11],[12]. A role for thalamic inputs has been proposed [11],[23], and both intracortical or thalamocortical synaptic inputs can eventually start up states [15],[23],[24]. However, it is known that the thalamus is not required for the rhythm to occur, since it persists after thalamic lesions and it can be recorded in isolated cortical slabs *in vivo* [25] and in cortical slices *in vitro* [18]. In the isolated cortex, it has been proposed that up states start by spontaneous spikes that activate the recurrent cortical circuitry, bringing the network to an up state where activity reverberates [19]. This model relies on strong cortical recurrence plus activity-dependent hyperpolarizing currents that terminate the up states and maintain down states. Alternative proposed mechanisms are the initiation of up states by summation of spike-independent stochastic releases of neurotransmitters or noise producing random transition between up and down states [26]. Those mechanisms would determine the initiation of up states given that they overcome the ones believed to start, and to maintain, down states such as potassium currents [17]–[19], metabolically modulated currents [20], or cortical disfacilitation [27].

The study presented here suggests that some of those seemingly mutually exclusive mechanisms regulating up and down states could indeed coexist. The analysis of intracellularly recorded up and down transitions by means of a reduced Langevin equation reveals that the stochasticity of up state occurrence varies with the dynamic state of the *in vivo* network. While in deep anesthesia, the occurrence of up states is not well fitted by the Langevin equation. Given that the Langevin equation describes the stochastic dynamics of a network, the bad fit to the data in deep anesthesia suggests that the process is not stochastic but deterministic and therefore controlled by non-random processes. However, *in vivo* during lighter anesthesia the time spent in the up and down states was better described by a one-dimensional model. In particular, the time spent in the down state was reasonably described by the model. This could indicate a role for random fluctuations in shaping the transitions from the down to the up-state. It is important to notice however that there are several aspects of the intracellular data that are not well described by the model (nor intended to be well described). There are for example high frequency oscillations in the upstate not captured by the model. Our findings suggest that different network mechanisms inducing up states that have been proposed by different authors and appeared to be incompatible, could indeed be simultaneously participating but in different functional states of the network. Thus, transitional states between sleep and awake (or light anesthesia) would be dominated by mechanisms involving stochasticity while deep sleep would be dominated by deterministic mechanisms. Another possible scenario is that in which the same mechanisms of initiation of up states would trigger more or less regular waves. This possibility has been achieved in a computer model by varying the cortical synaptic strength [28].

## Methods

### Ethics statements

Rats were cared for and treated in accordance with the EU guidelines on protection of vertebrates used for experimentation (Strasbourg 3/18/1986) as well as local ethical guidelines and regulations.

### Fokker-Planck description

In this section, we describe how to extract the *effective* energy function from a data set like, for example, the temporal sequence of firing activity in a network of spiking neurons. We also show how to estimate the intensity of the concomitant noise. By doing this, we will able to write the stochastic neurodynamical equation describing the generation of the data set. We assume that the system is stationary.

#### One-dimensional Langevin equation

To keep the analysis as simple as possible, we focus on cases where the description of the neural system can be captured by one single dimension. In those cases, it suffices to project the original dynamical variables on the first principal component, that is, on the direction where the variables show highest variability. We denote this projection by 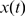. To derive the effective energy function underlying the stochastic evolution of 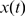, we assume that the trajectory 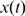 can be described by a one-dimensional Langevin equation generating a Brownian motion of a highly damped particle in a one-dimensional potential 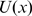 [29]

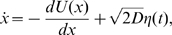
(6)where 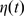 is a Gaussian fluctuation term with
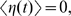
(7)

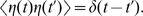
(8)The parameter 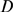 is the noise intensity. The time coordinate we use is dimensionless and normalized to the time constant, so that time derivatives are of order unity and noise intensities have dimensions of a variance. The probability density of the stochastic process described by Equations (6)–(8) satisfies the Fokker-Planck equation:
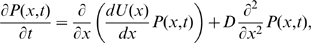
(9)where 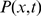 is the probability density of the random variable 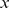. Equation (9) admits a nontrivial stationary solution given by
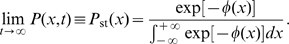
(10)where 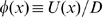 is the normalized potential. This solution is well-defined as long as the integral in the denominator converges, which is the case when the potential 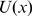 grows to infinity as 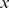 goes to 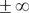. This limit is satisfied if we impose the condition that 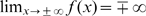.

#### Estimation of the one-dimensional potential

The effective potential 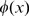 is obtained by fitting the estimated probability density of the reduced empirical data with the stationary solution, Equation (10). We perform this estimation assuming a continuous piecewise quadratic potential. First, we partition the range of 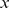 into 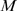 subintervals 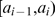, 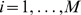. In each interval the potential is given by a quadratic polynomial
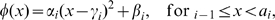
(11)where the 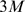 parameters 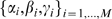 are to be determined. To ensure continuity and differentiability of the potential at the boundaries the subintervals, the parameters 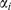 and 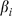 must obey the recurrent relation
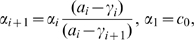


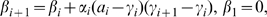
for 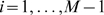. We are then left with 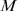 free parameters, which we denote by 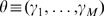. Given the experimental data 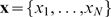, the values of the free parameters 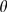 are set maximizing the logarithm of the likelihood function
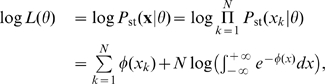
(12)where we assume in the second equality that the data is drawn independently and identically from the distribution 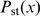, given by Equation (10). The maximization over 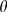 is computed using a downhill simplex method [30],[31].

#### Estimation of the noise intensity

In addition to the potential 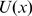 the effective stochastic dynamical equation (6) and the Fokker-Planck counterpart, Equation (9), involve the noise intensity 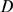. This parameter has to be specified in order to describe the full stochastic system. We estimate 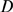 through the mean escape time of the system from a metastable state. The analytical form of the mean escape time from an interval 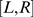 for the stochastic process described by Equation (6) starting at 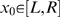 is [29] (an alternative procedure was derived in [32] for this case using piecewise parabolic potential profiles):
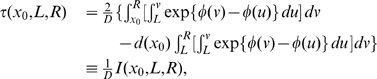
(13)where in the first equality we have defined
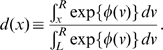
The mean escape time 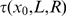 can be estimated from empirical data, while 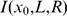 can be evaluated numerically once the potential has been approximated maximizing Equation (12). If we denote by 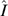 and 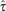 the estimated values of 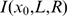 and 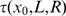, the noise intensity can be inferred from the relation (13),
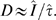
(14)


Once the energy function 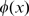 and the noise intensity 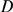 are determined, the effective description given by Equations (6)–(8), or, equivalently, by the associated Fokker-Planck Equation (9), is complete. Note that 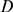 fixes also the time scale of the problem. The stationary distribution of the data determines uniquely the normalized potential 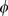 but it does not specify the time scale of dynamical evolution. Only after fixing 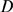 can we also fit the time scale of the data. In fact, the estimation of the residence time distributions shown in Results was carried out by explicit simulations of the Langevin Equation (6), using the maximum-likelihood value of the noise intensity 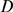 that fits best the mean escape time 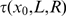 from a particular interval 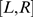, given the initial condition 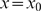.

### Large-scale spiking network

#### Population rates

The firing rate of population 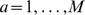 is defined as 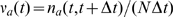, where 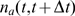 is the total number of spikes emitted in population 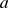 between 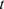 and 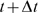, being 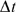 a small time interval, and 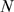 is the number of neurons in the population.

#### Probability densities

The 2-dimensional probability density function for the population rates 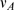 and 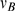, 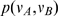, shown in Figure 4, was estimated from 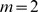 trials of 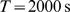 simulated time each, and using a timestep of 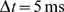. This gives a total of 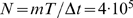 data points per trial. The duration of the trial was long enough for the network to to alternate between the two decision states a few tens of times. Such transitions were due to finite-size effects in the network, and allowed the system to explore most of the state space within the whole duration of the trial. We then invoked ergodicity, by which time averages are identified with state space averages, and estimate the probability density from the sample 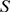 formed by all the visited states 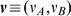 across all trials
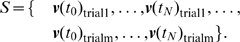



#### Reduction to one dimension

The dimensionality of the system can be further reduced by projecting the multidimensional data 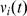 on the direction with the maximum variance, using principal component analysis (see, e.g., [33]). For all the cases considered, this direction coincided with the direction of the line connecting the two peaks of the estimated two-dimensional probability density. The one-dimensional histograms shown in Figures 4B,D result from projecting the original two-dimensional data points in 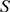 onto the principal component. Specifically, if the principal component is represented by the unit vector 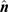, the sample of scalar values used in those histograms is 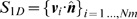). The histogram was clearly bimodal, with a trough at 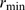.

#### Escape boundaries

We considered that a good estimate of the separatrix between two attractor basins was given, in the two-dimensional phase space, by the line passing through the origin and the point 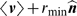, where 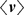 denotes the sample average. The inner escape boundaries used to estimate the noise intensity were also straight lines parallel to the separatrix and located at 0.75 the distance between the separatrix and the peaks. Thus, the inner boundary for peak 1 was a line parallel to the separatrix and passing through the point 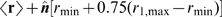, where 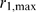 is the value of 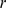 at which the one-dimensional histogram showed the local maximum corresponding to peak 1. The inner boundary for peak 2 was found analogously.

### Intracellular recordings in auditory cortex of the anesthetized rat

Recordings from primary auditory cortex A1 were obtained from adult wistar rats (230–350 g). Anesthesia was induced by intraperitoneal injection of ketamine (100 mg/kg) and xylacine (8–10 mg/kg). The animals were not paralyzed. Supplemental doses by intramuscular injection of ketamine were 75 mg/(kg h) and were given with intervals of 30–60 min. The depth of anesthesia was monitored by the recording of low-frequency electroencephalogram (EEG) and the absence of reflexes. The anesthesia level was deeper after a new dose and would progressive lightened during the interval (see Results). Rectal temperature was maintained at 37, heart rate (250–300 bpm) and blood 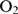 concentration (95%). Once in the stereotaxic apparatus, a craniotomy (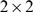 mm) was made at coordinates AP −3.5 to 5.5 mm from bregma, L 7 mm. After opening the dura, intracellular recordings were obtained with borosilicate glass capillaries 1 mm O.D. 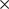 0.5 I.D. (Harvard Apparatus) filled with potassium acetate (resistances 50–80 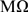). For stability, and to avoid desiccation, agar (4%) was used to cover the area. Data was acquired with a CED commercial acquisition board (Cambridge Electronic Design, UK) and its commercial software Spike 2. Further details of the procedure can be found in [15].

## Supporting Information

Text S1 Description of the network of spiking neurons used to generate synthetic data.
(0.09 MB PDF)
