# Supplementary material for: Effective Reduced Diffusion-Models: A Data Driven Approach to the Analysis of Neuronal Dynamics
Source: PLoS Comput Biol. 2009 Dec 4;5(12):e1000587. doi: 10.1371/journal.pcbi.1000587 (PMC2778141; doi:10.1371/journal.pcbi.1000587)
Supplement: Text S1 — Description of the network of spiking neurons used to generate synthetic data. (0.09 MB PDF) [file pcbi.1000587.s001.pdf]

Supplementary information

Region of bistability for the one-dimensional rate model

For a given value of a, the endpoints of the interval $[\theta_l, \theta_r]$ where the system is bistable are found from the bifurcation condition

$$f(x) = 0 \quad \text{(fixed point)},$$
$$f'(x) = 0 \quad \text{(bifurcation)}.$$

This corresponds to finding the values of θ where the bifurcation diagram (x^*, θ) has a limit point. The interval turns out to be

$$[\theta_l, \theta_r] = \left[\ln\left\{(1/y_- - 1)e^{ay_-}\right\}/a, \ln\left\{(1/y_+ - 1)e^{ay_+}\right\}/a\right],$$

where

$$y_\pm \equiv (1 \pm \sqrt{1 - 4/a})/2.$$

The stability diagram 1D is just the plot of the interval of bistability as a function of a.

Network of spiking neurons showing bistability

We used the network introduced by Brunel and Wang [1], in its particular implementation as a two-choice decision-making network [2].

Network

The network contains $N_E = 800$ pyramidal cells (excitatory) and $N_I = 200$ interneurons (inhibitory). Excitatory neurons are divided in three subpopulations: $p = 2$ *selective* populations (of $0.15 N_E$ cells each, called A and B in the article), whose neurons encode the two possible choices to make, and a population containing the remaining, non-selective neurons ($0.7 N_E$). The network is fully connected. Neurons within a selective population are strongly coupled, by a factor $w_+ > 1$ above the baseline connection weight, while neurons from different selective populations are weakly connected, with $w_- < 1$. Connections involving non-selective cells are set to baseline level, $w = 1$. To keep the mean recurrent excitatory weight constant for different values of w_+, w_- has to be set to $1 - f(w_+ - 1)/(1 - f)$.

External inputs

To model spontaneous background activity, every neuron in the network is coupled through $N_{\text{ext}} = 800$ synaptic connections to an external source of Poisson-distributed, independent spike trains of rate 3 Hz. The presence of stimuli is modeled by an increase λ in the rate of spikes afferent to the cells in the associated selective population. The total spike rate received by every cell is thus $\nu_{\text{ext}} = 2400\,\text{Hz} + \lambda_{A,B}$, where λ_A and λ_B refer to the specific inputs to cells in populations A and B respectively. For non-selective and inhibitory neurons $\lambda = 0$. For convenience we define the overall input increase in both populations $\lambda = (\lambda_A + \lambda_B)/2$ and the bias $\Delta\lambda = (\lambda_A - \lambda_B)/2$.

Spiking Dynamics

The model neuron is a leaky integrate-and-fire (LIF) cell, described in the following. The subthreshold membrane potential V of a LIF neuron evolves according to

$$C_m \frac{dV(t)}{dt} = -g_m(V(t) - V_L) - I_{\text{syn}}(t),$$

where C_m is the membrane capacitance (see numerical values in table 1), g_m is the membrane leak conductance, V_L is the resting potential, and I_{syn} is the synaptic current.

The synaptic current includes glutamatergic excitatory components (mediated by AMPA and NMDA receptors) and inhibitory components (mediated by GABA). External cells contribute to the current only trough AMPA receptors. The total current is given by

$$I_{\text{syn}}(t) = I_{\text{AMPA,ext}}(t) + I_{\text{AMPA,rec}}(t) + I_{\text{NMDA}}(t) + I_{\text{GABA}}(t).$$

with the different currents defined by

$$I_{\text{AMPA,ext}}(t) = g_{\text{AMPA,ext}}(V(t) - V_E) \sum_{j=1}^{N_{\text{ext}}} s_j^{\text{AMPA,ext}}(t),$$

$$I_{\text{AMPA,rec}}(t) = g_{\text{AMPA,rec}}(V(t) - V_E) \sum_{j=1}^{N_E} w_j s_j^{\text{AMPA,rec}}(t),$$

$$I_{\text{NMDA}}(t) = \frac{g_{\text{NMDA}}(V(t) - V_E)}{1 + \gamma \exp(-\beta V(t))} \sum_{j=1}^{N_E} w_j s_j^{\text{NMDA}}(t),$$

$$I_{\text{GABA}}(t) = g_{\text{GABA}}(V(t) - V_I), \sum_{j=1}^{N_I} s_j^{\text{GABA}}(t)$$

where w_j are the synaptic weights, s_j^x is the fraction of open channels for each receptor, and g^x is the synaptic conductance for receptor $x = $ AMPA, NMDA, GABA. The values for the synaptic conductances and the reversal potentials V_E and V_I are given in table 1. NMDA currents are voltage dependent and controlled by the intracellular magnesium concentration ($[\text{Mg}^{2+}] = 1$ mM), with parameters $\gamma = [\text{Mg}^{2+}]/(3.57\text{ mM}) = 0.280$ and $\beta = 0.062\,(\text{mV})^{-1}$.

The fraction of open channels in cell j, for all receptors, is described by the following differential equations:

$$\dot{s}_j^{\text{AMPA,ext}}(t) = -s_j^{\text{AMPA,ext}}(t)/\tau_{\text{AMPA}} + \sum_k \delta(t - t_j^k - \delta),$$

$$\dot{s}_j^{\text{AMPA,rec}}(t) = -s_j^{\text{AMPA,rec}}(t)/\tau_{\text{AMPA}} + \sum_k \delta(t - t_j^k),$$

$$\dot{s}_j^{\text{NMDA}}(t) = -s_j^{\text{NMDA}}(t)/\tau_{\text{NMDA,decay}} + \alpha x_j(t)(1 - s_j^{\text{NMDA}}(t)),$$

$$\dot{x}_j(t) = -x_j(t)/\tau_{\text{NMDA,rise}} + \sum_k \delta(t - t_j^k - \delta),$$

$$\dot{s}_j^{\text{GABA}}(t) = -s_j^{\text{GABA}}(t)/\tau_{\text{GABA}} + \sum_k \delta(t - t_j^k - \delta),$$

where the rise time constant for NMDA currents is $\tau_{\text{NMDA,rise}} = 2$ms, and $\alpha = 0.5\text{ ms}^{-1}$; rise time constant for AMPA and GABA currents are neglected. Decay time constants for AMPA, NMDA, and GABA synapses are $\tau_{\text{AMPA}} = 2$ ms, $\tau_{\text{NMDA,decay}} = 100$ ms, and $\tau_{\text{GABA}} = 10$ ms. The sums over k represent a sum over spikes emitted by pre-synaptic neuron j at time t_j^k. Transmission delay is set to $\delta = 0.5$ ms in all channels.

References

1. Brunel N, Wang X (2001) Effects of neuromodulation in a cortical networks model of object working memory dominated by recurrent inhibition. J Comput Neurosci 11: 63–85.

2. Wang XJ (2002) Probabilistic decision making by slow reverberation in cortical circuit. Neuron 36: 955–968.

Table 1. Parameters used in the network of integrate-and-fire neurons.

Parameter	Value	
**Network parameters**:		
N_E: number of excitatory cells	800	
N_I: number of inhibitory cells	200	
N_{ext}: number of cells in the external module	800	
p: number of selective populations	2	
f: fraction of exc. cells in a particular selective population	0.15	
w_+: relative strength of single potentiated synapses	1.9	
ν_{ext}: spike rate at external synapse	2.4 kHz	
**Neuronal parameters**:	**excitatory**	**inhibitory**
V_L: leak reversal potential	-70 mV	
V_{thr}: firing threshold	-50 mV	
V_r: reset potential	-55 mV	
C_m: membrane capacitance	0.5 nF	0.2 pF
g_m: membrane leak conductance	25 nS	20 nS
V_E/V_I: resting potential (excitatory/inhibitory)	0 mV	-70 mV
τ_{rp}: refractory period	2 ms	1 ms
**Synaptic parameters**:	**excitatory**	**inhibitory**
δ: transmission delay	0.5 ms	0.5 ms
$g_{\text{AMPA,ext}}$:	2.08 nS	1.62 nS
$g_{\text{AMPA,rec}}$: recurrent AMPA synaptic conductance	104 pS	81 pS
g_{NMDA}: recurrent NMDA synaptic conductance	327 pS	258 pS
g_{GABA}	1250 pS	973 pS
γ: modulatory factor of magnesium blocking	0.280	
β: gain factor in magnesium blocking	1/(16.1 mV)	
τ_{AMPA}	2 ms	
τ_{GABA}	10 ms	
$\tau_{\text{NMDA,decay}}$	100 ms	
$\tau_{\text{NMDA,rise}}$	2 ms	
α	0.5 ms^{-1}	
